# Recognizing thyrotoxicosis in a patient with bipolar mania: a case report

**DOI:** 10.1186/1744-859X-7-3

**Published:** 2008-02-19

**Authors:** Catherine See-Ning Lee, Burton Hutto

**Affiliations:** 1 Department of Psychiatry, University of North Carolina, Chapel Hill, NC, USA; 2 Dorthea Dix Hospital, Raleigh, NC, USA

## Abstract

**Background:**

A thyroid stimulating hormone level is commonly measured in patients presenting with symptoms of mania in order to rule out an underlying general medical condition such as hyperthyroidism or thyrotoxicosis. Indeed, many cases have been reported in which a patient is initially treated for bipolar mania, but is later found to have a thyroid condition. Several case reports have noted the development of a thyroid condition in bipolar patients either on lithium maintenance treatment or recently on lithium treatment.

**Case presentation:**

We review a case in which a patient with a long history of bipolar disorder presents with comorbid hyperthyroidism and bipolar mania after recent discontinuation of lithium treatment.

**Conclusion:**

Physicians should consider a comorbid hyperthyroidism in bipolar manic patients only partially responsive to standard care treatment with a mood stabilizer and antipsychotic.

## Background

Multiple cases of patients with thyrotoxicosis presenting with symptoms clinically indistinguishable from bipolar mania have been reported [1-8]. Moreover, lithium for the management of preexisting bipolar disorder has many known effects on thyroid function [1,2,4,6,8-10]. We report the case of a patient whose chronic lithium was discontinued owing to other concerns and who then presented with manic and psychotic symptoms apparently related to thyrotoxicosis.

## Case presentation

Ms F is a 59-year-old female with a long history of bipolar disorder, previously well controlled on a stable dose of lithium carbonate, who presented for hospitalization with an apparent manic episode. She reported four weeks of decreased sleep, hypersexuality, mood lability, increased spending, impulsive behavior and psychotic symptoms. The patient and her sister both noted that the symptoms were consistent with her manic episodes in the remote past. Ms F reported a recent change in her medication from lithium carbonate to aripiprazole, made by a new psychiatrist, who was uncomfortable with prescribing lithium after the patient had been hospitalized for lithium toxicity a month prior to admission.

Her psychiatric history included multiple episodes of mania with subsequent hospitalizations in the remote past. Past medication trials included risperidone, aripiprazole, quetiapine, lithium carbonate and depakote. The past medical history was significant for arthritis, hypercholesterolemia, irritable bowel disorder and a history of depressed thyroid-stimulating hormone (TSH), with normal free T4. On transfer from an outside psychiatric hospital, her medications were quetiapine 50 mg at night, simvastatin 40 mg daily, lithium citrate 300 mg twice daily and aspirin 81 mg daily. On initial admission to the outside psychiatric hospital, Ms F's medications were aripiprazole 15 mg at night, simvastatin 40 mg daily and aspirin 81 mg daily.

Physical examination determined that she was overweight with findings of 3+ deep tendon reflexes bilaterally, a fine tremor noted in bilateral upper extremities, mild proptosis, a non-tender, non-palpable thyroid, non-pitting edema in her lower extremities and dry skin.

Mental status examination determined that she was alert and oriented ×4, fairly groomed in appropriate dress with good eye contact, and polite and cooperative with the interview and exam. Her speech was not pressured, loud or rapid. She denied auditory and visual hallucinations, suicidal ideation and homicidal ideation. However, she did endorse paranoid ideation and delusions with racial themes. Her thought processes were mostly linear, with some circumstantiality, but no flight of ideas, no looseness of associations or ideas of reference. Ms F's cognition and memory were intact.

Her admission laboratory tests including complete blood count, chemistry, liver function tests, rapid plasma reagin (RPR) and urine drug screen were within normal limits, with the exception of TSH level of less than 0.005 (0.60–3.30 μIU) and free T4 level of 2.11 (0.71–1.40 ng/ml). TSH and free T4 from an outside psychiatric hospital two days prior to admission were 0.008 and 2.48, respectively.

Once admitted to an acute psychiatric unit, Ms F was continued on lithium 300 mg twice daily. Her dose was increased to a total daily dose of 900 mg after a lithium level of 0.4 mmol/l on hospital day 4. Risperidone 2 mg nightly for the treatment of psychotic symptoms was added to her quetiapine 25 mg nightly. Repeated physical examinations showed return to normal deep tendon reflexes and resolution of tremor by hospital day 5, but her TSH level was still less than 0.005 on day 5 with a free T4 level of 1.81. Owing to continued psychosis and insomnia, on day 7 her risperidone was increased to 3 mg nightly and zolpidem 10 mg nightly replaced the quetipine. No changes were made to patient's lithium dose after day 4 because her lithium level was stable at 0.9 units by day 8. On day 11 her TSH level remained at less than 0.005, with a free T4 level of 1.57. On day 12 the risperidone was increased to 3 mg nightly with 0.5 mg daily. She had not returned to her baseline by day 12, despite a therapeutic level of lithium and a moderate dose of risperidone. On day 13, her zolpidem was discontinued and quetiapine was restarted at 100 mg nightly for management both of insomnia and psychotic symptoms. A free T3 level of 4.4 (0.2–0.4 ng/dl) substantiated a diagnosis of hyperthyroidism on day 13. Methimazole 10 mg nightly was started, and the patient reported her first night of good sleep.

According to Ms F and available collateral information, her TSH level had been depressed for more than three years, with a normal free T4 level. Consequently, she remained untreated throughout that time, followed with laboratory tests every six months. However, given the patient's persistent symptoms of insomnia and psychosis, despite adequate doses of risperidone and lithium, and given her initial presentation with symptoms of clinical hyperthyroidism including proptosis, a tremor and increased deep tendon reflexes, there was a high index of suspicion for thyrotoxicosis. Thus, the patient's TSH and free T4 were followed throughout her hospitalization. Treatment was withheld initially owing to potential hypothyroidism secondary to restarting lithium. The patient was ultimately started on methimazole for treatment of hyperthyroidism, which was diagnosed after serial TSH levels continued to be depressed, with an elevated free T3 level measured on hospital day 13. Ms F responded well to the start of methimazole, with rapid resolution of insomnia and psychotic symptoms. Both the patient and her family members noted a return to baseline by hospital day 19, after six days of stable treatment on methimazole, lithium, quetiapine and risperidone.

The patient's refractory symptoms suggest that patients may in fact present with comorbid hyperthyroidism and bipolar mania. Another interpretation of the patient's partial response to therapeutic levels of lithium and adequate doses of risperidone is that her hyperthyroidism had been unmasked by the discontinuation of lithium, and that her presentation was entirely the result of a rebound type reaction [9]. In other words, the removal of lithium, an agent known to suppress thyroid activity through several mechanisms, unveiled the patient's previously controlled hyperthyroidism [3,9]. However, given the patient's history and family collateral confirming that her behavior was consistent with manic episodes in the past, it is more likely that the lithium served the dual purpose of thyroid suppression and treatment of her bipolar disorder. Thus, the patient's resulting mania after discontinuation of her lithium was exacerbated by a concurrent hyperthyroid state/silent thyroiditis.

## Conclusion

We have reported a case in which a patient who was stable on lithium for decades was taken off of it owing to toxicity and then presented with manic and psychotic symptoms.

Several mechanisms have been described regarding how lithium disrupts the hypothalamic-pituitary-thyroid axis, thus leading to thyroid dysfunction [1,2,4,6]. Transient hypothyroidism has been observed in up to 30% of patients treated with lithium [9]. A literature review by Carmaciu et al [3] yielded 50 cases of lithium-associated hyperthyroidism after a reduction or discontinuation of lithium treatment. Although the majority of lithium-treated patients that develop abnormal thyroid conditions present with hypothyroidism, several cases of lithium-associated thyrotoxicosis, from a silent thyroiditis to Graves' disease, have been reported [9,11].

Lithium is a monovalent cation that becomes concentrated in the thyroid gland where it interferes with the release of thyroid hormone in several ways [5]: inhibition of adenylate cyclase stimulated by TSH in normal patients; decreased thyroid iodine uptake; decreased release of T4 and T3; decreased hepatic metabolism of T4 to T3; and increased intrathyroidal iodine stores through iodine retention.

Carmaciu et al [3] further delineates five mechanisms in which lithium is thought to mediate thyrotoxicosis in some patients that have been on maintenance treatment: (1) lithium triggered autoimmune process with resultant anti-thyroid antibodies; (2) abnormal iodine kinetics, that is, overflow of thyroid hormone after expansion of the intrathyroid iodine pool; (3) Jod-Basedow-like phenomenon; (4) direct toxicity to thyroid follicules, resulting in release of thyroglobulin; and (5) coincidental Graves' disease and hyperthyroidism.

## Competing interests

The author(s) declare that they have no competing interests.

## Authors' contributions

Both authors have read and approved the final manuscript. CSNL was the primary provider for the patient described in above, and wrote the initial draft of the report. BH reviewed and supervised the writing of the case.
